# Use of Bone Bank Grafts in Revision Total Hip Arthroplasty: Patient Characteristics at a Referral Center

**DOI:** 10.3390/medicina61071246

**Published:** 2025-07-10

**Authors:** Thiago de Carvalho Gontijo, Luiz Octávio Pereira Xavier, Lucas Carneiro Morais, Gustavo Waldolato Silva, Janaíne Cunha Polese, Raquel Bandeira da Silva, Amanda Aparecida Oliveira Leopoldino

**Affiliations:** 1 Faculdade Ciências Médicas de Minas Gerais, Belo Horizonte 30150-270, Brazil; lucmorais10@hotmail.com (L.C.M.); janainepolese@hotmail.com (J.C.P.); raquel.silva@feluma.org.br (R.B.d.S.); amanda.leopoldino@cienciasmedicasmg.edu.br (A.A.O.L.); 2 Hospital Universitário Ciências Médicas de Minas Gerais, Belo Horizonte 30150-270, Brazil; octaviomed@hotmail.com (L.O.P.X.); gwaldolato@hotmail.com (G.W.S.)

**Keywords:** total hip arthroplasty, revision total hip arthroplasty, epidemiology, bone allograft, tissue banks

## Abstract

*Background and Objectives*: To characterize the epidemiological profile of patients undergoing revision total hip arthroplasty (THA) using bone allografts from a tissue bank, and to identify clinical and surgical factors associated with the selection of graft type in cases of severe periprosthetic bone loss. *Materials and Methods*: This observational, cross-sectional study involved a retrospective review of medical records from a specialized referral center, including revision THA procedures performed between 2013 and 2019. Data were collected on 36 variables covering demographic details (age, sex), surgical history of both hips, comorbidities, medication use, perioperative complications, hospitalization, surgical technique, and characteristics of the bone grafts used. Patients were grouped based on the type of allograft received—structured or morselized (impacted)—and comparative analyses were performed. *Results*: A total of 67 revision THA cases were evaluated, with a mean patient age of 63.2 years. Nearly half (47.8%) had no prior hip revision. The average number of previous procedures per patient was 1.73, and the mean interval from primary THA to revision was 178.4 months. Morselized bone allografts were used in 66.7% of cases, and structured allografts in 33.3%. Patients receiving structured grafts had undergone a significantly higher number of prior surgeries (*p* = 0.01) and had a longer duration since the initial THA (*p* = 0.04). *Conclusions*: These findings suggest that younger patients undergoing primary total hip arthroplasty may be at increased risk for complex revision procedures involving structured grafts later in life, underscoring the need for long-term monitoring and tailored surgical planning in this population.

## 1. Introduction

It is estimated that approximately 630,000 hip replacement procedures were performed in the United States in 2017 [1]. Projections indicate that the number of primary total hip arthroplasty (THA) procedures will increase by 176%, with revision total hip arthroplasty (THAR) procedures expected to rise by 43% to 70% between 2014 and 2030 [2]. Total hip arthroplasty revision is a highly complex surgical procedure with high costs for the health system [2,3]. In addition to the impact on the quality of life [4] and work productivity of these patients, revision THA also places a burden on the social security system [5,6].

Aseptic loosening is often the main cause of revision in long-term arthroplasties [7]. Its association with bone defects is common, and its treatment is a challenge for medical staff, in addition to major defects being related to worse surgical results [8]. The mechanisms underlying prosthetic loosening and the development of periprosthetic bone defects are progressively being elucidated [9,10]. Although the clinical presentation of prosthetic loosening varies, with some patients presenting severe bone loss and others minimal compromise, our study focuses on identifying clinical and surgical factors related to the severity of bone defects and the selection of grafting techniques.

The integration of advanced technologies such as three-dimensional (3D) printing, rapid prototyping, and finite element modeling has led to significant advancements in preoperative planning, implant customization, and postoperative outcomes in orthopedic surgery. Traditional bone grafting procedures, although effective, are often limited by donor site morbidity, restricted material availability, and suboptimal anatomical fit. In this context, 3D printing offers a promising solution by enabling the fabrication of patient-specific bone grafts with high anatomical precision and tailored mechanical properties [11]. The use of biocompatible materials and scaffold designs further enhances osteointegration and accelerates healing. Additionally, rapid prototyping based on computed tomography (CT) data provides tangible and accurate physical models of the patient’s anatomy, allowing for detailed surgical simulations, improved spatial understanding, and reduced intraoperative uncertainty [12]. These models also serve as valuable educational resources and communication tools for multidisciplinary teams.

Complementing these physical modeling strategies, finite element analysis (FEA) has become indispensable in orthopedic biomechanics. By simulating the distribution of stress and strain within joint structures under physiological loading, FEA offers critical insights into joint function, degeneration, and prosthetic performance [13]. Computational optimization studies have shown that even subtle refinements in prosthetic component geometry can significantly improve load distribution and enhance initial implant stability. Göktaş, H. et al. [14], for example, employed topology optimization for acetabular reconstruction and demonstrated that optimized geometries effectively reduce peak interface stresses and promote more uniform load transfer to the host bone. These findings underscore the fundamental importance of adequately restoring acetabular bone stock—whether through impacted or structural grafts—as a prerequisite for achieving biomechanical conditions favorable to optimal implant function. Proper bone support enables the implant to perform as intended, facilitating osteointegration and increasing prosthesis longevity.

THAR surgery using a tissue bank bone graft is a treatment for failure of a previous arthroplasty associated with bone defects. It has been used with good results for decades [15]. However, most of the published studies are regarding techniques and outcomes [9,10,16]. Epidemiological data on patients undergoing revision arthroplasty remains limited [3], with most available information focusing primarily on primary joint replacements. However, there has been a growing recognition of the importance of systematic data collection, as evidenced by the progressive establishment of National Arthroplasty Registry programs worldwide over the past decades [1,17].

Given these gaps in the literature, the present study aims to expand the epidemiological understanding of patients undergoing revision THA with bone allografts. Specifically, it seeks to identify clinical factors associated with graft selection and arthroplasty failure in cases involving extensive bone defects.

## 2. Materials and Methods

This is a cross-sectional study with data collection and analysis coming from electronic medical records of patients from a referral hospital which were found to be undergoing THA revision with reconstruction using a bone bank graft. The work was approved by the ethics committee of the Ciências Médicas of Minas Gerais institution under the number (CAAE-14177519.2.0000.5134), and the use of the informed consent form was waived. Two researchers with clinical and research expertise conducted data collection. Prior to initiating the review process, evaluators underwent structured training encompassing the operational definitions of all 36 variables, standardized data abstraction protocols, and systematic navigation procedures within the electronic medical record system. The training protocol incorporated a pilot phase utilizing anonymized clinical cases to assess interrater reliability and promote consistency in data interpretation across evaluators. Following independent data extraction, the researchers conducted an interrater reliability assessment using Cohen’s kappa and the intraclass correlation coefficient (ICC), which demonstrated high consistency and confirmed the robustness of the data collection process [18].

The quantitative variables collected included patient age, interval between THA and revision using bone allografts, number of previous surgeries, time since the most recent surgery, number of comorbidities and medications, length of stay in the intensive care unit and overall hospitalization, volume of blood transfused, time from initial clinical evaluation to revision surgery, and number of postoperative complications. Postoperative complications were analyzed separately for the hospitalization period and the early postoperative phase (up to 90 days), beyond which data were excluded due to significant sample attrition. In addition, dichotomous variables were recorded, including sex, place of residence, graft type, surgical laterality, history of complications, presence of comorbidities, contralateral total hip arthroplasty, smoking status, and alcohol use, among others.

The endorsed medical records constitute all patients who underwent THAR with bone graft from a tissue bank, being duly documented between 2013 and 2019. This period corresponds to the beginning of the performance of the surgical team with this type of procedure and conducted by a senior surgeon. All documented cases during this period were included in the study, with no additional inclusion or exclusion criteria applied regarding age, general health status, or comorbidity profile. This approach ensured that the sample accurately reflected the full population treated with this technique at our referral center.

Descriptive statistics were used to characterize the sample, as well as percentages to analyze the variables in terms of frequency. The Pearson′s or Spearman’s correlation coefficient were used to verify the correlations between the selected variables [19]. We also considered the analysis of two main groups within the sample, those which used structured grafts or other impacted morselized bone allograft with correlations with each other through the T-test. The significance level was set at 0.05, and 95% confidence intervals were calculated in all the statistical tests.

The choice between Pearson’s and Spearman’s correlation coefficients was based on the distribution of the variables. Pearson’s was used for normally distributed variables, while Spearman’s was applied for non-parametric data. Normality was assessed through descriptive statistics and histogram visualization. For group comparisons between patients receiving structured versus impacted bone grafts, independent samples t-tests were conducted. The assumptions of normality and homogeneity of variances were verified using visual inspection and Levene’s test, respectively.

## 3. Results

### 3.1. Clinical and Demographic Profile of the Study Population

A total of 67 hips were included in the analysis. Table 1 presents the demographic and clinical characteristics of the study population. The majority of patients were male (53.7%), with a mean age of 63.2 years (range: 33–88 years). Hypertension was the most prevalent comorbidity (53.7%); metabolic disorders such as obesity and hypothyroidism were present in 42.3% of the patients with other comorbidities (11 out of 26 patients), indicating that these conditions were the most frequent among the comorbidities evaluated beyond hypertension and diabetes. The mean number of comorbidities per patient was 1.16, while the average number of home medications was 1.61. Tobacco use and alcohol consumption were uncommon, with 84.6% and 76% of patients, respectively, denying these dependencies.

Osteoarthritis was the predominant underlying condition (74.6%). A total of 32 patients (47.8%) had undergone only one prior THA on the affected side, with an overall mean of 1.73 previous procedures. Regarding laterality, 45 patients (67.2%) had not undergone total hip arthroplasty of the contralateral limb at the time of the revision procedure. Implant loosening was the leading cause of revision surgery, accounting for 89.6% of cases.

Regarding surgical timing, the mean interval between the primary THA and the revision procedure using bone allograft was 178.4 months. The average duration between the initial orthopedic evaluation and the revision surgery was 32 months. Bone grafting was performed without additional reconstruction in 42 patients (66.7%). The majority of patients (61.2%) did not experience in-hospital complications, and 86.4% were free of early postoperative complications (within 90 days). Blood transfusions were required in only 26.9% of cases. The mean length of hospital stay was 11.94 days, with an average intensive care unit (ICU) stay of 2.03 days. Eight patients (12.9%) had positive intraoperative cultures, and 11.9% developed acute surgical site infections within 90 days. Two deaths were reported: one due to in-hospital complications and another due to early postoperative complications.

### 3.2. Comparative Analysis of Clinical Variables According to Graft Type

To further investigate the clinical and surgical factors that may influence the selection of bone graft type, the sample was stratified into two groups: patients who received structured allografts and those who received impacted morselized allografts. A comparative statistical analysis was conducted to identify significant associations between graft type and key clinical variables, including the number of previous surgeries, time since the last surgical procedure, and the interval between the primary THA and the revision procedure requiring grafting.

The analysis revealed that patients in the structured graft group had undergone a significantly greater number of prior surgeries compared to those in the impacted graft group (*p* = 0.02). Additionally, the interval between the primary THA and the revision arthroplasty was significantly longer among those receiving structured grafts (*p* = 0.04). These findings suggest that the use of structured grafts may be more frequently indicated in cases of more advanced bone loss and complex surgical histories. No significant difference was observed between the groups regarding the time elapsed since the most recent surgical intervention (*p* = 0.86). Table 2 summarizes the comparative analysis between graft types and the corresponding clinical variables.

In addition to statistical significance, we assessed the clinical relevance of our findings using Cohen’s *d*. The difference in the number of previous surgeries between patients receiving structured and impacted bone grafts demonstrated a moderate effect size (*d* = 0.65), reinforcing that structured grafts are more commonly used in cases with greater surgical complexity. Similarly, the difference in time since the primary THA showed a moderate effect size (*d* = 0.59), suggesting that longer implant survival before revision may be associated with more advanced bone loss necessitating structural grafts. In contrast, the non-significant difference in time since the last surgery had a negligible effect size (*d* = −0.05), indicating limited clinical relevance. These findings support the interpretation that surgical history, particularly the number of prior procedures and duration since primary arthroplasty, are robust indicators of graft type selection.

## 4. Discussion

The present study identified a relatively younger group of older adults, with a mean age of 63.2 years, undergoing revision total hip arthroplasty with bone allografts, and a low average number of comorbidities (1.16 per patient). This profile is relevant given that many patients are of working age, with substantial life expectancy remaining in a country where average longevity is 75.5 years [20].

Notably, the mean age observed in our cohort is lower than that reported in broader U.S. national databases for THAR, where the average age is approximately 67.4 years [21]. Similar findings are observed in cohorts that are more specific. Yu et al. [3], in a study conducted at a high-volume referral center in the United States involving patients who underwent multiple revision procedures, reported a mean age of 59.6 years. Vastel et al. [22] described an average age of 60.2 years in a revision series utilizing bone grafts, involving patients with a mean of 3.8 previous hip surgeries. Likewise, Zazgyva et al. [23] reported a mean age of 60.3 years in patients undergoing revision THA with structured bone grafts, although in that case series, no prior revisions were recorded.

These findings raise the hypothesis of a possible trend wherein patients undergoing multiple hip arthroplasty revisions tend to be younger. A plausible explanation is that clinical practice favors performing primary THA in older patients, who may not survive long enough to require revision. While this may explain the younger age at revision in our cohort, it also underscores the need for careful interpretation when comparing our findings with studies involving patients with multiple revisions, who may present with different clinical trajectories and risk profiles. Furthermore, our sample may reflect referral bias, as all the included patients were treated at a tertiary care center specializing in complex revision arthroplasty cases with extensive bone loss, often necessitating the use of bone bank allografts.

It is well established that THA procedures are increasingly performed in younger patient populations [24]. Several studies have reported that younger age at the time of primary THA is associated with a higher risk of implant failure and a greater likelihood of requiring revision surgery [25,26]. However, a key question that remains to be elucidated is whether younger age at the time of primary arthroplasty also contributes to a higher incidence of multiple revisions, given that THAR is known to have a shorter implant survival compared to primary THA [27].

In the present study, the mean time elapsed between primary THA and the revision procedure was approximately 15 years, and the mean age at the time of revision was 63.2 years. These findings suggest that many patients underwent their initial arthroplasty at approximately 48 years of age. This age is notably lower than the reported national average for primary THA in the United States, which stands at 67.4 years [21]. Such a discrepancy reinforces the importance of long-term follow-up in younger THA recipients, as their extended life expectancy increases the probability of prosthetic failure and the eventual need for one or more revision procedures. Further research is warranted to determine whether early primary THA independently contributes to the likelihood of undergoing multiple revisions and to better understand the long-term outcomes of this patient subgroup.

**Figure 1:**
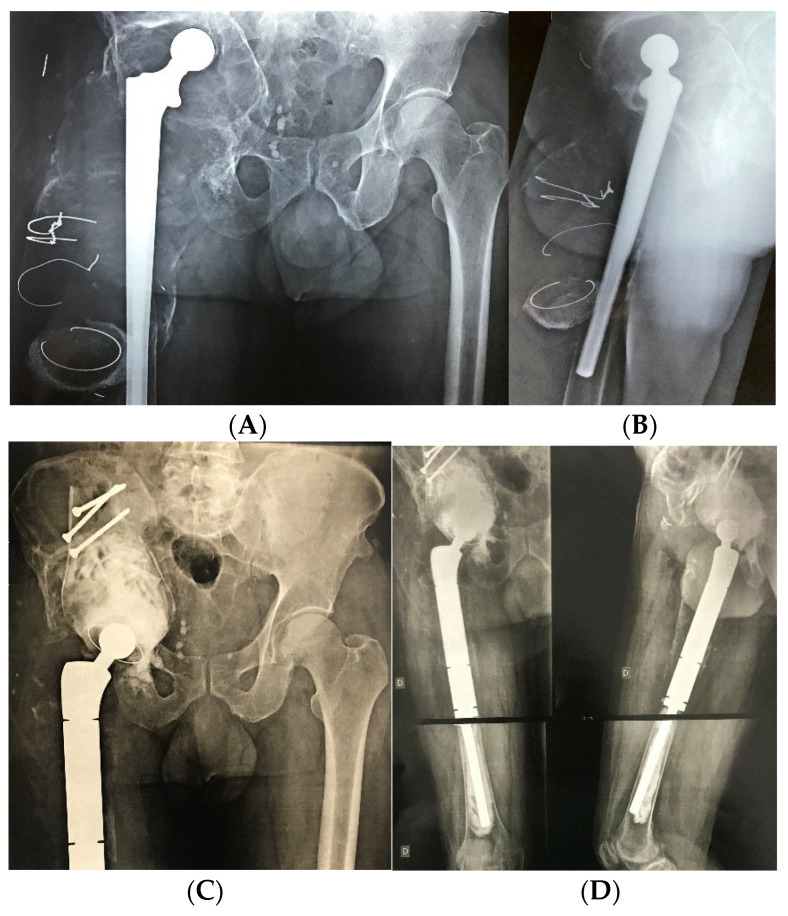
Preoperative upper image of a large acetabular and femoral bone defect, with proximal migration of the femoral and distal shaft of the acetabulum (**A**,**B**). Lower images of the post-operative reconstruction with structured graft from the distal femur in the pelvis (**C**,**D**). (D: right side, Author’s personal source).

**Figure 2:**
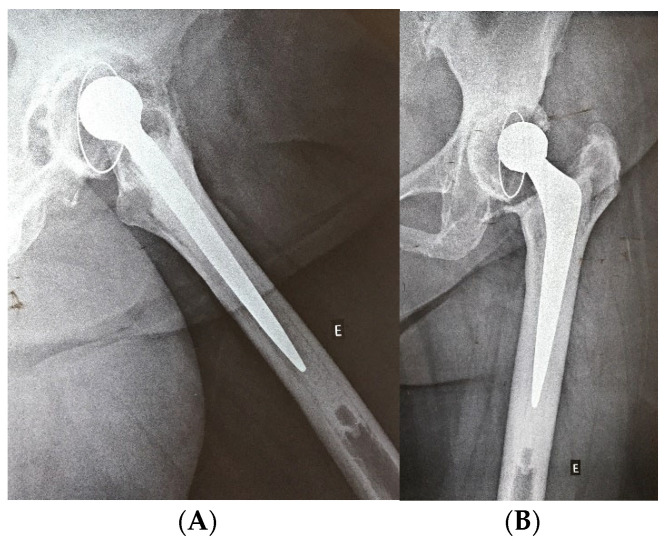

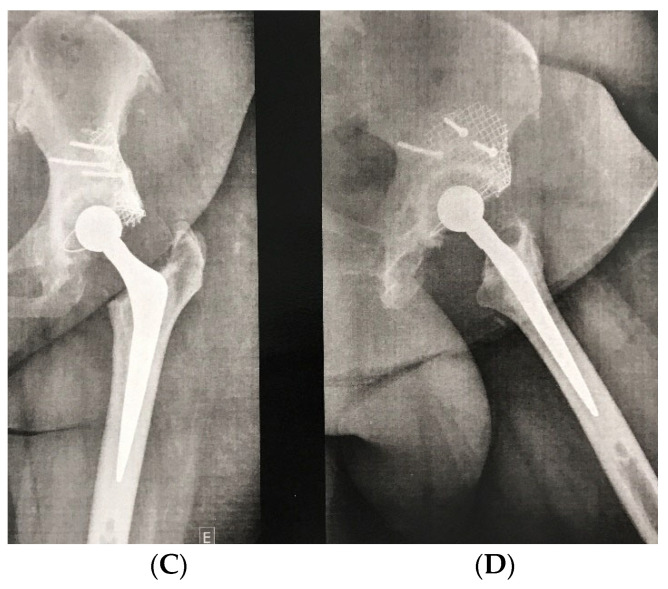
Preoperative upper image of a minor acetabular bone defect, with loosening of the acetabular component (**A**,**B**). Lower postoperative images of THAR with only acetabular reconstruction using impacted bone graft (**C**,**D**). (E: left side, Author’s personal source).

Prosthetic loosening was identified as the most frequent complication leading to revision surgery, occurring in 89.6% of the cases. All the patients included in this study presented with periprosthetic bone defects, in accordance with the inclusion criteria, and consequently required the use of bone allografts sourced from a tissue bank. The pathophysiological mechanisms underlying bone defect formation in the context of implant loosening are being increasingly clarified in the literature [9,10,28]. However, the specific factors that predispose certain patients to develop extensive bone loss, while others with similar loosening present only minimal defects, remain insufficiently understood. In the context of THAR, the choice of bone allograft technique is typically guided by the size and morphology of the bone defect. Structured allografts are generally indicated for the reconstruction of extensive and non-contained defects, particularly those lacking cortical support (Figure 1) [29]. In contrast, impacted morselized bone grafts are more commonly employed in the management of contained or smaller cavitary defects (Figure 2) [30]. Further details on the Paprosky classification of acetabular bone loss and its correlation with graft type are provided in the Supplementary Materials (Figure S1 and Table S1). 

From a biomechanical standpoint, the distinction between structural, stable and fragmented, unstable, or comminuted stabilization in bone grafting is fundamental to interpreting both experimental and clinical outcomes. The mechanical environment established by the chosen stabilization technique, whether it provides rigid fixation or permits micromotion, directly influences bone healing dynamics and the reproducibility of biomechanical studies. The literature evidence consistently highlights that mechanical stability can exert a more pronounced effect on healing outcomes than biological variables, genetic background, or therapeutic interventions. This perspective reinforces the importance of graft type selection as a key element in the clinical success of revision arthroplasty procedures involving bone defects.

Given the established relationship between the morphology of bone defects and the type of graft used in THAR, we stratified our sample into two groups: patients who received structured allografts and those treated with impacted morselized bone grafts. Our analysis demonstrated that the use of structured grafts was associated with a significantly longer interval between the primary THA and the subsequent revision procedure. This finding may indicate a tendency for primary THA to be performed at a younger age in patients who subsequently required structured grafting. This interpretation aligns with existing literature, which has consistently shown that younger age at the time of primary THA is associated with a higher risk of revision and implant failure [25,26,31]. Moreover, there remains a lack of robust longitudinal data regarding the outcomes and durability of revision procedures in younger patients [27], especially those undergoing multiple revisions. It is plausible that the duration between the primary and revision procedures is not, in itself, the principal driver of prosthetic wear and failure. Rather, the younger physiological age at the time of the index arthroplasty may play a more critical role [32].

Additionally, studies have indicated that the biological response to polyethylene wear particles may be more pronounced in younger individuals, potentially accelerating periprosthetic bone loss [33]. Within this context, our findings suggest a possible association between younger age at primary THA and the development of extensive or complex bone defects that ultimately necessitate the use of structured allografts during revision. Another significant correlation identified in our analysis was the association between the use of structured bone allografts and a higher number of previous surgical procedures on the affected hip. This finding suggests that patients requiring structured grafts often had more complex surgical histories, potentially resulting from an initial THA performed at a younger age [25,26], a recognized risk factor for revision procedures. Consequently, the presence of large bone defects, necessitating structured grafts, appears to be positively associated with multiple prior interventions on the same joint.

From a clinical perspective, the increased risk of earlier revision in younger patients should not serve as a contraindication to performing THA, nor should it limit their access to surgical treatment and its associated benefits. As highlighted in the literature, advancements in surgical techniques and implant technology may mitigate some of these long-term risks [24]. However, it is essential that patients are appropriately informed about these risks, and such factors should be carefully considered in the shared decision-making process. Our findings reinforce the notion that THA in younger patients should not be regarded as a definitive solution but rather as the beginning of a long-term clinical pathway. This highlights the importance of implementing structured follow-up programs aimed at early detection of implant wear or mechanical failure. Such monitoring would allow timely interventions to prevent the progression to large, complex bone defects, which are associated with increased technical difficulty, higher morbidity, and poorer clinical outcomes [34]. While the treatment of bone defects in revision THA is supported by extensive literature, including the use of bone allografts, other biological materials, and emerging technologies [16,29], further research is needed to better understand the factors contributing to the development of complex bone defects and strategies to prevent them. These efforts are crucial given the technical challenges and adverse outcomes often associated with managing extensive bone loss in revision arthroplasty [34].

This study has several limitations. Its retrospective design introduces inherent biases, and the analysis was dependent on the quality and completeness of data recorded in medical charts. We attempted to minimize these issues through researcher training and interexaminer reliability analysis during data collection. Nevertheless, certain valuable variables, such as body mass index, pain intensity, symptom duration, operative time, detailed bone defect classifications, and differentiation between isolated acetabular or femoral revisions, were excluded due to insufficient or inconsistent documentation. Additionally, the sample is subject to selection bias, as it was composed exclusively of patients referred to a tertiary center for complex revision cases involving severe bone loss and therefore does not reflect the broader epidemiological profile of revision THA, which more commonly involves technically fewer demanding procedures without graft use. Furthermore, implant traceability was not feasible due to the absence of a national arthroplasty registry, and no analysis service was available for the explanted components. All the patients had metal-on-polyethylene articulations, most likely utilizing conventional polyethylene liners, as these are standard in the Brazilian public healthcare system and do not include enhancements such as crosslinking.

It is important to note that this study was not specifically designed to identify predictive factors for the development of complex bone defects. Future studies are warranted to explore these variables in greater depth. Additionally, the establishment of a national arthroplasty registry would be a critical step toward collecting standardized, high-quality data that can support more robust analyses and ultimately improve patient outcomes.

One important limitation of this study is the absence of long-term clinical outcome data, such as postoperative functionality, return to activities, and patient-reported quality of life measures. Due to the retrospective design and variability in clinical documentation and follow-up duration, these outcomes could not be consistently collected for the entire cohort. The lack of standardized functional data limits a more comprehensive assessment of the clinical effectiveness of the procedures performed. Furthermore, it is important to note that the study was conducted exclusively at a tertiary referral center specializing in complex revision cases, resulting in a sample composed of patients with advanced periprosthetic bone loss and more challenging surgical profiles. This selective inclusion limits the generalizability of the findings to other settings, particularly those managing less technically demanding revision procedures. Future prospective studies with systematic follow-up and broader inclusion criteria are needed to provide more representative and generalizable results.

## 5. Conclusions

This study offers a comprehensive characterization of patients undergoing THAR with bone allografts, elucidating key clinical and surgical factors associated with the selection of structured versus impacted morselized grafts. The data indicate that the use of structured allografts is significantly correlated with a greater number of previous surgeries and a longer interval between the primary and revision procedures. These associations suggest that younger age at the time of primary THA may predispose patients to more extensive periprosthetic bone loss over time, ultimately necessitating more complex reconstructive strategies. Such findings emphasize the importance of long-term clinical monitoring in patients who receive THA at an earlier age. Proactive surveillance and timely intervention in these cases may mitigate the progression to severe bone defects, thereby reducing surgical complexity and associated morbidity. Despite the limitations inherent to its retrospective design and potential selection bias toward more complex surgical cases, this study reinforces the critical role of continuous follow-up in optimizing patient outcomes. Additionally, the results highlight the need to strengthen national arthroplasty registries to enhance implant traceability, support evidence-based clinical decision-making, and improve the overall quality of data available for research. Ongoing investigations into the etiopathogenesis of extensive bone defects and the biological integration of grafts are essential to developing more effective and durable solutions in revision hip arthroplasty.

## Figures and Tables

**Table 1:** Characteristics of the participants.

Characteristic	n = 67
Age, mean SD	63.2 (10.9)
Sex, male—n (%)	36 (53.7)
Region, metropolitan—n (%)	35 (52.2)
Bone allograft, impacted bone graft—n (%)	42 (66.7)
Basic pathology, arthrosis—n (%)	50 (74.6)
Previous surgeries, only primary total hip arthroplasty—n (%)	32 (47.8)
Previous complications—loosening—n (%)	60 (89.6)
Systemic arterial hypertension, yes—n (%)	36 (53.7)
Diabetes mellitus, no—n (%)	60 (89.6)
Other comorbidities, metabolic diseases—n (%)	11 (42.3)
Total hip arthroplasty opposite member, no—n (%)	45 (67.2)
Smoking, no—n (%)	55 (84.6)
Alcoholism, no—n (%)	50 (76.9)
Hospital complications, no—n (%)	41 (61.2)
Operative infection, no—n (%)	59 (88.1)
Late complications, no—n (%)	57 (86.4)
Late operative infection, no—n (%)	61 (92.4)
Growth intraoperative cultures, no—n (%)	54 (87.1)
Blood transfusion, no—n (%)	49 (73.1)

**Table 2:** Comparison between graft type regarding the studied variables.

Variable		Mean (SD)	Mean Difference (SD) [CI 95%]	*p*-Value	Cohen’s *d*
Previous surgery (n)	Structured	2.14 (0.91)	0.55 (0.21) [0.11 to 0.98]	0.02	0.65
	Impacted bone graft	1.60 (0.78)			
Last surgery (months)	Structured	126.63 (87.99)	−3.61 (20.77) [−45.16 to 37.95]	0.86	−0.05
	Impacted bone graft	129.24 (68.71)			
Date of primary total hip arthroplasty	Structured	214.53 (119.16)	53.40 (24.76) [3.85 to 102.96]	0.04	0.59
	Impacted bone graft	161.12 (71.76)			

SD: standard deviation; CI: confidence interval; *p*: significance.

## Data Availability

The data is available from the corresponding author upon reasonable request.

## Supplementary Materials

The following supporting information can be downloaded at: https://www.mdpi.com/article/10.3390/medicina61071246/s1, Figure S1. Paprosky Classification of Acetabular Bone Loss [35]: (A) Type 1, (B) Type 2A, (C) Type 2B, (D) Type 2C, (E) Type 3A, (F) Type 3B. Table S1. Summarizes the correlation between the Paprosky classification, defect type, and the most commonly used type of bone graft. References [36,37] are cited in the supplementary materials.

## Abbreviations

CI	Confidence Interval
ICU	Intensive Care Unit
SD	Standard Deviation
THA	Total Hip Arthroplasty
THAR	Revision Total Hip Arthroplasty
