# Supplementary material for: Use of Bone Bank Grafts in Revision Total Hip Arthroplasty: Patient Characteristics at a Referral Center
Source: Medicina (Kaunas). 2025 Jul 10;61(7):1246. doi: 10.3390/medicina61071246 (PMC12299342; doi:10.3390/medicina61071246)
Supplement: Supplementary file 1 [file medicina-61-01246-s001.zip › medicina-3687778-supplementary.pdf]

Supplement – Correlation Between Paprosky Classification and Type of Bone Graft Used in Revision Total Hip Arthroplasty

The Paprosky classification is one of the most widely used systems to categorize patterns of acetabular bone loss in revision total hip arthroplasty (THA). This classification is based on the evaluation of pelvic radiographs, considering the migration of the acetabular component and the integrity of key structures such as the acetabular roof, medial wall (represented by the teardrop), Kohler's line, and the ischium. Based on this assessment, defects are categorized into Types 1, 2 (2A, 2B, 2C), and 3 (3A, 3B), indicating increasing degrees of bone loss and structural compromise.

The relationship between defect type and the need for bone grafting has been well described in the literature since the original study by Paprosky et al. (1994) (1), and has been corroborated by subsequent studies (2,3). In general, particulate grafts are preferred for cavitary defects with preserved bone structure (Types 1 and some Type 2), whereas structural grafts are indicated for segmental or global defects with compromised bone support (particularly Types 2B, 3A, and 3B).

In Type 3A defects, there is superolateral component migration greater than 2 cm and significant loss of the lateral acetabular roof, particularly between the 10 and 2 o'clock positions. The absence of this superolateral support hinders stable fixation of the acetabular component and prevents adequate press-fit with hemispherical implants. In such cases, structural bone grafts, such as distal femur or proximal tibia segments, are more appropriate to restore bony continuity and provide a stable foundation for implant fixation.

In Type 3B defects, the damage is even more extensive, with superomedial migration and widespread destruction of the acetabular columns, especially between 9 and 5 o'clock. The term "femur sculpted in the shape of the number 7" refers to a structural graft obtained from the proximal femur segment, cut in the coronal plane. The natural curvature of the femoral head, neck, and calcar forms a configuration resembling the number 7, which allows reconstruction of the deficient acetabular contour. This graft is fixed to the pelvis using reconstruction plates and screws, providing mechanical stability until osseointegration occurs.

The illustration below (adapted from Telleria & Gee, 2013)(2) depicts the Paprosky classification types:

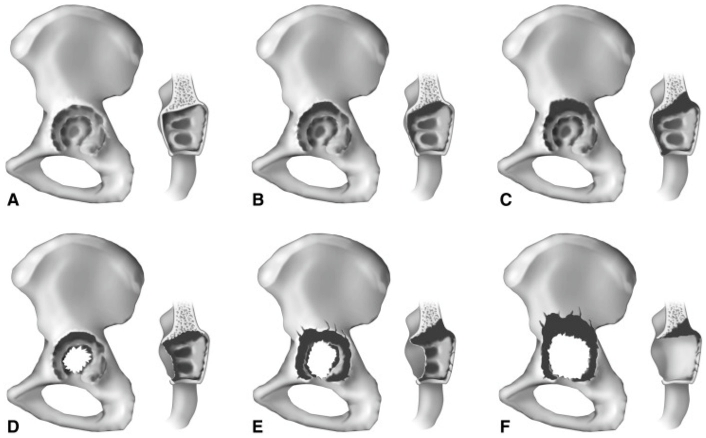

Figure S1. Paprosky Classification of Acetabular Bone Loss: (A) Type 1, (B) Type 2A, (C) Type 2B, (D) Type 2C, (E) Type 3A, (F) Type 3B. Adapted with permission from Paprosky WG, Perona PG, Lawrence JM. J Arthroplasty. 1994;9(1):33–44.

Table S1. Summarizes the correlation between the Paprosky classification, defect type, and the most commonly used type of bone graft:

**Paprosky Type**	**Main Defect Characteristics**	**Most Common Bone Graft Type**
Type 1	Minimal bone loss, lateral roof preserved	Particulate
Type 2A	Mild superior migration, intact dome	Particulate or none
Type 2B	Superolateral migration, compromised lateral roof	Structural (proximal femur)
Type 2C	Medial migration, compromised medial wall	Particulate (femoral head)
Type 3A	>2 cm superolateral migration, absent lateral roof	Structural (distal femur/tibia)
Type 3B	Superomedial migration, compromised columns	Structural (femur sculpted in number 7)

References:

1. Paprosky WG, Perona PG, Lawrence JM. Acetabular defect classification and surgical reconstruction in revision arthroplasty. A 6-year follow-up evaluation. J Arthroplasty. 1994;9(1):33–44.
2. Telleria JJM, Gee AO. Classifications in brief: Paprosky classification of acetabular bone loss. Vol. 471, Clinical Orthopaedics and Related Research. 2013.
3. Yu R, Hofstaetter JG, Sullivan T, Costi K, Howie DW, Solomon LB. Validity and reliability of the paprosky acetabular defect classification hip. Clin Orthop Relat Res. 2013;471(7).
